# Benzene: A critical review on measurement methodology, certified reference material, exposure limits with its impact on human health and mitigation strategies

**DOI:** 10.5620/eaht.2024012

**Published:** 2024-04-05

**Authors:** Poonam Kumari, Daya Soni, Shankar G Aggarwal

**Affiliations:** 1 CSIR-National Physical Laboratory, Dr. K.S. Krishnan Marg, New Delhi-110012, India; 2 Academy of Scientific and Innovative Research (AcSIR), Ghaziabad-201002, India

**Keywords:** Benzene, Certified reference materials, Standard measurement methods of benzene, Exposure limits

## Abstract

Benzene is a carcinogenic pollutant with significant emission sources present in the atmosphere. The need for accurate and precise measurement of benzene in the atmosphere has become increasingly evident due to its toxicity and the adverse health effects associated with exposure to different concentrations. Certified reference material (CRM) is essential to establish the traceability of measurement results. The present review compiles the available national and international measurement methods, certified reference materials (CRMs) for benzene and the limit of benzene in fuel composition (v/v) worldwide. Overall, the review indicates the benzene level in the atmosphere and the resulting impacts on the environment and human health, which frequently exceed the exposure limits of different environment regulatory agencies. An extensive literature review was conducted to gather information on monitoring and analysis methods for benzene, revealing that the most preferred method, i.e. Gas Chromatography- Flame Ionization Detector and Mass Spectrometry, is neither cost-effective nor suitable for real-time continuous monitoring. By analysing existing literature and studies, this review will shed light on the understanding of the importance of benzene pollution monitoring in ambient air and its implications for public health. Additionally, it will reflect the mitigation strategies applied by regulators & need for future revisions of air quality guidelines.

## Introduction

Benzene, Toluene, Ethylbenzene, and Xylenes, collectively known as BTEX, are highly hazardous aromatic group of toxic pollutants among all volatile organic compounds (VOCs). The International Agency for Research on Cancer (IARC) classifies “Benzene” as a known human carcinogen [1-6]. Benzene is also included in the list of 12 pollutants of the National Ambient Air Quality Standard (NAAQS) 2009) [7] that has an annual exposure limit 5 µ g/m3. Benzene is a colourless and highly flammable liquid widely used in various industries, including petrochemicals, rubber, and paint manufacturing [8-11]. It is also a component of gasoline and tobacco smoke [12]. Traffic emissions and fuel stations are predominant sources of benzene in urban areas [13-14]. It is a VOC that can evaporate into the air, and exposure can occur through inhalation of ambient air or direct contact with contaminated soil or water. Acute exposure to benzene can cause myeloid leukaemia, dizziness, headaches, nausea, and unconsciousness [15-24]. Long-term exposure to small amounts of benzene can lead to bone marrow damage, reduced red and white blood cell counts and anaemia, development of leukaemia, lymphoma, and other haematological cancers [17,25-26]. Additionally, exposure to benzene during pregnancy can cause birth defects [27]. Due contribution towards the formation of SOA (secondary organic aerosol) [28-35], and Ozone Formation Potential [36], benzene can have a negative impact on environment. Therefore, in urban air pollution, benzene stands out as a significant characteristic of aromatic hydrocarbons and has become a high-priority target for evaluation in ambient air. Continuous monitoring of toxic pollutants in the environment is necessary for maintaining a clean and safe environment. It also aids in protecting the health of the population and can serve as an executive tool for implementing prevention and control measures [37]. To protect public health, WHO (World Health Organization) and other regulatory agencies have established exposure limits for benzene in ambient air and at workplace. Although occupational exposure limit to benzene levels remains high in some countries and industries, it poses a significant risk to workers. Therefore, monitoring benzene levels in ambient air and other workplaces is essential to protect public health. Various monitoring techniques and technologies have been developed for accurate measurement of benzene discussed in this article. Real-time monitoring, active and passive sampling methods have their advantages and limitations. Their selection depends on the specific monitoring objectives and available resources.

Accurate calibration of benzene measurement instruments is essential to establish the measurement traceability of results obtained [38-40]. Accurate and precise data of benzene in the atmosphere is need of our due to its toxic nature and adverse health issues even exposure to very low concentrations. The accurate and traceable measurement is essential to support high-quality scientific research, regulatory compliance, the protection of human health, risk assessment and environmental policy-making efforts to mitigate benzene pollution and its ecological effects.

This review article will discuss gravimetrically prepared calibration gas mixtures of benzene and their national and international intercomparison of certified reference material (CRM) of benzene, sampling and analysis techniques, exposure limits in air and at different occupational areas. The accurate and reliable measurement of benzene in ambient air is crucial for assessing associated health risks, raising awareness of exposure sources, developing mitigation strategies and implementing effective control regulations to protect the human health.

## Materials and Methods

### Properties of Benzene

The US Environmental Protection Agency (EPA) has identified 40 HAPs the most risky hazardous pollutants (through Section 112 of CAAA) (the Clean Air Act Amendments 1990). Benzene (C6H6) CAS no. 71432 at room temperature is light -yellow liquid or colourless. The nature of benzene is highly flammable and it has a sweet odour. The density of benzene at 20°C is 0.87 g/cm3 with a melting point of 5.5 °C. It evaporates into the air very quickly even at room temperature due to relatively less B.P 80.1 °C and a high vapour pressure of 9.95 kPa at 20 °C. Benzene may sink in the low area because its vapour is heavier than air. Benzene dissolves only slightly in water. Its solubility is 1.8 g per/litre at 25 °C and is mostly miscible with organic solvents. The stability of benzene in the vapor phase depends on climate change or many factors of pollutants present in the air (Wikipedia). Its Henry’s Law constant is 550 Pa.m3 /mol at 25 ° C, which means it has the capability to volatilize into the environments from surface water. Its degradation is mainly due to a reaction with hydroxyl radicals, with a rate constant of 1.2 × 10^–12^ cm^3^ molecule^–1^s^–1^ at 298 K.[Table t1-eaht-39-2-e2024012]

### Sources & Sinks of benzene in air

Around 50 years ago, Haagen-Smit studied in Los Angeles and found that organic compound produced by human activity are significant air pollutant present into atmosphere [41]. Benzene can be found in atmosphere as a result of both natural and anthropogenic. It is considered as the hazardous air pollutant and 60 % of total benzene emissions coming from mobile sources [42]. In 2015, Sahu et al. suggested that benzene source in ambient air are anthropogenic, biogenic and other secondary sources, all contributing to some extent [43]. In 2006, Srivastava et al. conducted a study and found that mobile sources associated with benzene and other volatile organic compounds were uniformly distributed in Mumbai urban area [44]. Tobacco smoke is considered as indoor source of benzene. In United States 50 % exposure to benzene is due to cigarette smoke or second-hand smoke. It has been observed that room with tobacco smoke have many times benzene level higher than normal [45]. The evaporation of solvents used in paints, cleaning products, and degreasers containing benzene can release into the air. There are numerous factors responsible for the exposure of benzene in the atmosphere, which can be mainly categorized as both natural and anthropogenic sources as given in Table 2.

Benzene is a major component of gasoline and crude oil. that is released from mobile sources i.e gasoline engines [12]. It is used in gasoline as a component to improve engine performance and acts as an octane booster as well as antiknock properties [46]. It is released during the fuel combustion in vehicles, making vehicle exhaust an important source of benzene in urban areas [47]. However, due to its toxic or carcinogenic effects on human health, the proportion of benzene in fuel has significantly decreased over the past few decades. It is regulated by Directive 2009/30/EC, which mandates that it must be < 1 % (v/v) [48]. Many industrial processes, such as petroleum refining, chemical manufacturing, and rubber and plastic production emit benzene into the air. National Centre for Biotechnology Information (NCBI) 2021 describes that it is widely used in the United State, its rank within top twenty chemicals for production [49]. During production, substantial amount is released into the atmosphere, since 1988 production continuously increasing reaching 20 million tonnes. In recent time, there has been growing demand for benzene and it is expected to continue grows strongly in the coming years. In 2022, production reached 60.28 million metric tonnes with forecast that benzene market will reach around 76.04 million metric tons by 2030 [50].

Benzene is commonly used for manufacturing several chemicals and solvents such as ethylbenzene, cumene, and aniline plastics, drugs, dyes, detergent and insecticides [51]. Its maximum usage is for production of ethylbenzene, (for styrene) cumene and cyclohexane [52]. The global annual production of benzene market is expected to increase by 3 % yearly. The country which are main consumers is China, USA and Western Europe with almost equal shares of 16-17 % [52]. Natural emission of benzene includes vegetation, oceans, microbial decomposition, wildfires and volcanoes [53-56]. In 2022, Dickinson et al observed benzene level ranging from 0.04 to 25 ppbv. in the wildfire smoke [57]. Bihałowicz et al. 2022 also studied the fire impact on air quality. Non methane volatile organic compounds are emitting from landfills in urban areas. The observed concentration of BTEX pollutants were lower at upwind sites as compared to downwind sites [58]. Benzene is produced during the decomposition of organic material in landfills and can be released into the air. Benzene is also found naturally in petroleum and is released into the air during oil and gas drilling and extraction.

**Figure 1.**
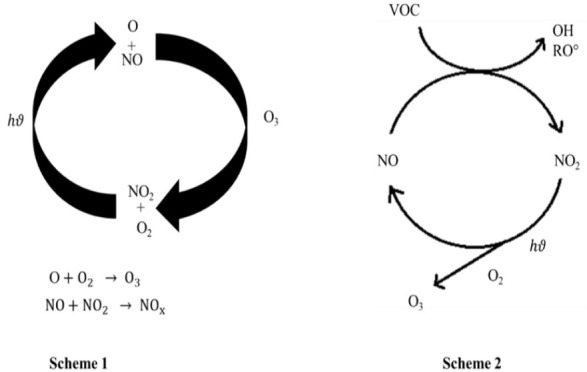
Chemistry of NOx and VOCs towards formation of Ozone in the environment.

VOCs involvement in physico-chemical processes at the troposphere is important in atmosphere, because these hydrocarbons participate in the formation of O3 and other photochemical activity [59]. In all VOCs, pollutants like BTEX increase the level of photochemical oxidants like ozone which is most harmful to both human beings and environment by making the smog in atmosphere [34,60]. The anthropogenic emission of NOx from industries, power plants burning of fossil fuels react with VOCs, produced secondary organic pollutants. The presence of NOx and VOCs in the environment is contributing to the formation of ozone, as indicated by the reactions in Fig. 1.

Benzene can undergo various chemical reactions, both in the environment and in industrial processes. These reactions can act as "sinks" for benzene, meaning they remove it from the environment. Benzene as a precursor participate in the formation of ozone and other secondary pollutants in ambient air [35]. Benzene and its substitute produced photochemical reactions in the presence of sunlight and hydroxyl radicals (OH), to form SOA and peroxyacetyl nitrate (PAN) [31-32,34].

Benzene can be degraded through reactions with hydroxyl radicals (OH°) produced hydroxy- cyclohexadienyl or OH-aromatic adduct, which is reactive species that help remove pollutants from the air. It can also be removed from water through various treatment processes, such as activated carbon adsorption or biological degradation.

### Fuel composition and its limits

The limits for benzene in fuel composition vary by country and region. Here, discussed benzene limits in gasoline for various parts of the world.

Benzene is a highly volatile organic compound (VOC), facilitating its use in gasoline as a component to enhance fuel combustion efficiency through improved vaporization. Benzene also has a high-octane rating, which means it can improve the engine's power and acceleration. However, the use of benzene in fuel has been linked to serious health effects, including an increased risk of cancer and other illnesses. As a result, various countries have implemented regulations to limit the amount of benzene in gasoline, and some have banned its use altogether. The Environmental Protection Agency has set limits of 1 % benzene by volume in gasoline sold in the United States, although some states have stricter limits. Also, the use of alternative fuel additives is made mandatory to reduce the need for benzene. The European Union (EU), China, Japan, Australia has set a limit of 1 % benzene and South Korea has set 0.5 % by volume in gasoline sold in the region.

In India, regulatory bodies of fuel qualities are MOP&NG i.e Ministry of Petroleum and Natural Gas, MoEF i.e Ministry of Environmental and Forest, MoRTH i.e Ministry of Road Transport and Highway and Bureau of Indian Standards (BIS). The benzene limit in gasoline is currently set at 1 % by volume. This limit was introduced by the BIS in 2016, as part of the Indian government's efforts to make better air quality and bring down adverse health effects associated with exposure to toxic pollutants. In 2001, Bharat Stage emission standard (BS II) norms described that benzene limit in gasoline 3 % by volume in metro cities and up to 5 % by volume nationwide. However, the Indian government recognized the need for more stringent regulations to keep safe public health and the atmosphere, particularly in urban areas where air pollution levels are high. BS III, IV and presently BS VI norms described 1 % limit of the benzene in gasoline [61].

It's worth noting that some states like Delhi in India have implemented even stricter limits on benzene in gasoline. Overall, the introduction of the 1 % benzene limit in Indian gasoline represents a positive step towards improving air quality and protecting public health. The Indian government continues to implement additional measures to reduce air pollution, including the promotion of alternative fuels and the introduction of vehicle emission standards. Some countries have lower limits for benzene in gasoline, particularly in regions with high air pollution or where the health risks associated with benzene exposure are well-established. In addition, some countries have implemented incentives or mandates to encourage the use of alternative fuel additives that reduce or eliminate the need for benzene in gasoline.

### Health effects of benzene

Loomis D and its group published a report in 2017, to determine the carcinogenicity of benzene. This investigation led to a meeting held in Lyon, France, where 27 scientists from 13 different countries participate at the IARC. During this meeting, they conclusively confirmed the carcinogenic nature of benzene, supported by substantial evidence. In several studies, it has been confirmed that chronic myeloid leukaemia and lung cancer observed with positive association with exposure of benzene [62]. Benzene, one of the most hazardous pollutants among BTEX (Benzene, Toluene, Ethyl benzene, Xylene) has been classified as Group one human carcinogen by both USEPA and IARC [5,63,64]. Ross D et al. 2000 and Snyder R. et al. 2004 studied that Benzene must be metabolized to became toxic or carcinogenic.

**Figure 2. f2-eaht-39-2-e2024012:**
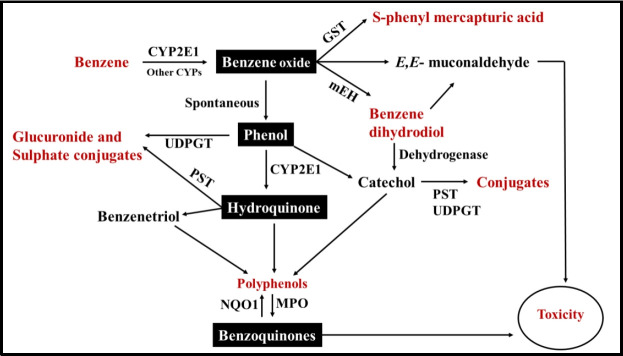
Scheme for metabolism of benzene

In 2010, Martyn T Smith have done detail study on metabolism of benzene and its carcinogenic health effects relevance [17]. Summarized scheme of metabolism of benzene as given in Fig 2.

Here, CYP2E1 denotes cytochrome P450 2E1; GST means glutathione-S-transferase; NQO1 represents NAD(P)H: quinone oxidoreductase 1; MPO means myeloperoxidase; UDPGT means uridine diphosphate glucuronyl transferase; PST denotes phenol sulfotransferase; mEH means microsomal epoxide hydrolase.

Long-term exposure to benzene level in environment has been linked to the development of leukaemia in human. In animals, exposure to benzene has been associated with leukaemia’s, lymphomas, various tumor types, delayed bone formation, and low birth weights. Additionally, bone marrow damage during pregnancy and affect biological human organs [17,26].[Fig f2-eaht-39-2-e2024012]

**Figure 3.**
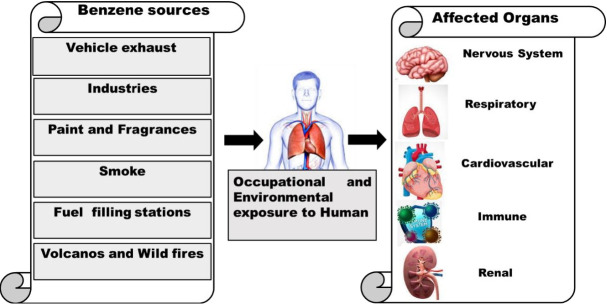
Emission sources of benzene and its biological human health effects.

A pictorial representation of emission sources and its affected biological organs of human is given, as shown in Fig. 3.

According to the findings reported by Bahadar et al. (2014), benzene exposure level ranging from 638.8 -5110.8 µ g/m^3^ in China, Italy, and Turkey may be linked to childhood leukemia, myeloid leukemia, myelodysplastic syndrome, and Non-Hodgen lymphoma [65-66]. Another study suggested that concentrations below 15.9 µ g/m^3^ of benzene, with longterm exposure, may lead to the defeat of hematological parameters [67,68]. In a study done by Lan et al. in 2004, the effects of low amount of benzene exposure on 240 workers were examined, revealing a decrease in platelet and WBC counts among 140 workers due to prolonged exposure to benzene concentration of 3.19 µ g/m^3^ [69]. Lupo et al. (2011) found that reproductive and chronic toxic effects were observed in association with benzene emissions, leading to birth defects [70]. Another study revealed pregnant woman exposed to benzene or other organic solvents may face an increased the risk of less birth weight and fetal malformations [71,72]. Furthermore, research has indicated that benzene fumes emitted from traffic-related air pollution may rise up the risk of adverse effects on infant weight growth [25,27]. So above studies concluded that exposure to benzene can lead to serious health issues, highlighting the importance of monitoring and regulating the exposure of benzene to protect public health and environments.

### Health risk methodology

**Figure 4.**
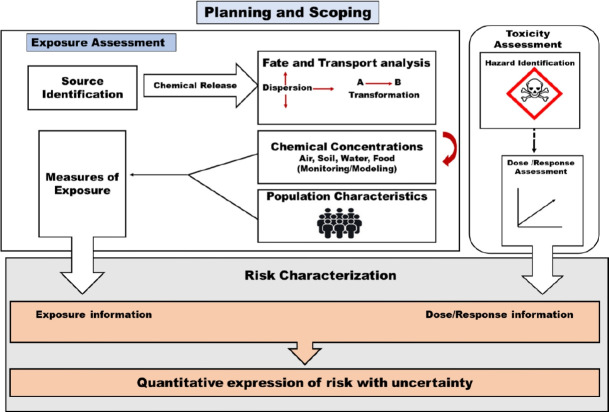
Health risk assessment process

A health risk assessment methodology incorporates to evaluate the influence of ambient benzene pollutant. Basic scheme follows to estimate the health risk assessment process as given in Fig 4.

According to the guidelines provided by USEPA, a health risk assessment was conducted to evaluate the inhalation intake of a contaminant present in the air. The assessment was based on the Risk Assessment Guidance for Superfund (RAGS), Part A [73], considering factors such as inhalation rate (IR) and body weight (BW). The risk associated with exposure to benzene in ambient air was estimated using the equations i.e Incremental Lifetime Cancer Risk (ILCR) and Hazard Quotient (HQ). To determine the ILCR value, the Lifetime Average Daily Dose (LADD) was estimated through inhalation pathway using the following Eq. (1)


(1) ILCR = LADD × CSF

Where LADD represents the Lifetime Average Daily Dose in milligrams per kilogram per day (mg/kg-day), and CSF stands for the cancer slope factor of benzene, which is 0.0273 mg/kg-day as provided in the Risk Assessment Information System [74].

The LADD for population was calculated using Eq. (2)


(2) $$\mathrm{LADD}=\frac{\mathrm{CA}\times \mathrm{CF}\times \mathrm{IR}\times \mathrm{EF}\times \mathrm{ET}\times \mathrm{ED}}{\mathrm{BW}\times \mathrm{AT}}$$

where,

CA (µ g/m^3^) denotes contaminant concentration in air,

CF is the Conversion Factor (1 mg/1000 μg, i.e., 0.001 mg/μg),

IR (m^3^/h) represents inhalation rate as per US EPA standard (20 m³/day, i.e., 0.83 m³/h)

ET (h/day) signifies exposure time in hours per day,

EF (days/years) represents exposure frequency,

ED (years) represents exposure duration,

BW (kg) denotes body weight (average body weight),

AT (day) represent averaging time (years× days/years)

CSF (mg/kg-day) is inhalation cancer slope factor

Additionally, the non-cancer risk was assessed using the Hazard Quotient (HQ) calculated through Eq. (3)


(3) $$\mathrm{HQ}=\frac{\mathrm{LADD}}{\mathrm{RfD}}$$

Where, the reference dose (RfD) value for benzene is 0.03 mg/kg-day [75].

In 2009, the Environmental Protection Agency (EPA) has adopted a revised approach for assessing human health risks related to inhaling contaminated air, using the Inhalation Dosimetry Methodology [76]. According to this method exposure concentrations (ECs) is calculated for individuals exposed to contaminants through inhalation, following EPA guidelines. The cancer and non-cancer risks associated with benzene pollutant via inhalation pathway are determined by estimating its EC value. In the Inhalation Dosimetry Methodology, the level of contaminants in the air measured in mg/m3 i.e denoted by EC used as exposure metric [76]. This approach contrasts with the method outlined in RAGS, Part A [73], which calculates inhalation intake based on inhalation rate (IR) and body weight (BW). The EC represents a time-weighted average concentration in ambient air over the duration of exposure.

Cancer risk (CR) due to benzene concentration inhalation is calculated as using Eq. (4)


(4) CR = EC × IUR

Here, CR represents the excess cancer risk, and IUR is the inhalation unit risk of benzene, given as 0.0000078 (μg/m^3^)-1 in the Risk Assessment Information System (RAIS) [74].

The EC of population is calculated using Eq. (5)


(5) $$\mathrm{EC}=\frac{\mathrm{CA}\times \mathrm{ET}\times \mathrm{EF}\times \mathrm{ED}}{\mathrm{AT}}$$

Where,

EC (µ g/m^3^) denotes exposure concentration,

CA (µ g/m^3^) denotes contaminant concentration in air,

ET (h/day) represent exposure time,

EF (days/week) is exposure frequency,

ED (weeks/exposure period) indicates exposure duration,

AT (hours/exposure period) represent averaging time,

IUR (µ g/m3)-1 signifies inhalation unit risk,

Hazard Quotient (HQ) is for non-cancer risk calculated using Eq. (6)


(6) $$\mathrm{HQ}=\frac{\mathrm{EC}}{\mathrm{RfC}\times 1000µg/\mathrm{mg}}$$

Where, RfC is the inhalation toxicity value for benzene i.e 0.03 mg/m^3^. (https://iris.epa.gov/AtoZ/?list_type=alpha)

The value (ILCR and CR) ˃1×10^-6^ was considered to have carcinogenic effects of concern, and a value ≤ 10^-6^ was considered an acceptable level The value of HQ ˃ 1 indicates adverse non-carcinogenic effects of concern, and the value of HQ ≤1 was considered an acceptable level [77].

Numerous studies have explored the cancer risk associated with benzene concentration in ambient air. Garg et al reported a probability range for benzene-related cancer risk of 0.50 estimated between 1.29×10^-6^ – 1.80×10^-5^ [78]. Additionally, Tabatabaei et al conducted a health risk assessment for children exposed to hookah smoke indoors. They observed ILCR associated with exposure in smoking and non-smoking households was estimated at 15× 10^-6^ and 1.8×10^-6^ respectively [79]. Mostly literature study exceeds the limit of cancer risk i.e 1× 10^-6^ given by WHO. In 2023, Poonam et al. estimates the cancer and non-cancer health risk associated due to benzene concentration observed in ambient air and at a fuel station in New Delhi. The average of seven months CR and HQ values of benzene exposure using Tenax sorbent tubes at a fuel station was found to be 9×10^-4^ ± 4×10^-4^ and 3.74 respectively. The ILCR value was found with 50% standard deviation due to benzene exposure in ambient [2-3].

### Ambient and occupational exposure limits of benzene

Annual exposure limit for benzene in ambient air worldwide as described countrywide. India, Lebanon, Russia, South Korean, New Zealand, North America Botswana, Albania prescribed annual limit as 5 μg/ m^3^; Iraq, Japan as 3 μg/m^3^; Scotland, Northern Ireland as 3.25 μg/m^3^; Israel as 1.3 μg/ m^3^; Syria as 20 μg/ m^3^; Vietnam, Morocco, South Africa, Belarus as 10 μg/ m^3^; France 2 μg/ m^3^; Malta and Sweden as 2 to 3.5 μg/ m^3^. [80]. In 2019, detail study of benzene standard reported by Sekar A.

In India, the CPCB has established a maximum permissible limit for benzene in the NAAQS 2009, which is 5 μg/m^3^ specified as annual time weighted average (TWA) [7].

In Directive 2022/431/EU occupational exposure limit value of the European Union specified as 1 ppm (3.25 mg/m³) over an 8-hour period. OSHA has specified occupational exposure limits for benzene with a Permissible Exposure Limit (PEL) of 5 ppm for short term exposure (STEL) over 15 minutes duration, and Threshold Limit Value (TLV) of 1 ppm as a TWA over an 8-hour period. The National Institute of Occupational Safety and Health (NIOSH) recommends STEL to 1ppm over15 minutes and a TLV-TWA of 0.1 ppm over an 8-hour period [81]. Similarly, American Conference of Governmental and Industrial Hygienists (ACGIH) set limit exposure to benzene as TWA of 0.5 ppm for 8-hour and STEL 2.5 ppm [2, 82]. Worldwide occupational and ambient exposure limits is given in Table 3 and 4.

### National Air quality standard of benzene

For developing countries, managing ambient air quality and minimizing adverse health effects due to air pollution from various sources is imperative. This is achieved through the implementation of "National Ambient Air Quality Standards" which is mandatory. In India, the CPCB initiated the first air quality standards as per Act 1981, Section 16(2) on November 11, 1982. These standards were revised in 1994. To raise awareness of the importance of air quality compliance under the Air (Prevention and Control of Pollution) Act 1981, CPCB established the National Ambient Air Quality Monitoring (NAMP) Network, covering 209 cities and towns across the country. CPCB also holds powers and functions for determining and controlling various air pollutants as per the Air Act 1981. Subsequently, in 1984, CPCB introduced the nationwide program "National Ambient Air Quality Monitoring Programme" (NAAQM), later revised as National Air Quality Monitoring Programme (NAMP). By 2015, the number of monitoring stations had increased to 612, covering almost 254 cities in the nation under the NAAQM scheme (https://www.cpcb.nic.in/). CPCB notified revised NAAQS in 2009 for air quality implementation. This revised NAAQS notification includes 12 major air pollutants, one of which is benzene, a highly hazardous pollutant. The annual limit prescribed by NAAQS (2009) for benzene is 5 µ g/m^3^, with no short-term standard specified. In India, except for benzene, no guidelines and standards have been defined for monitoring VOC pollutants in ambient air. Two methods for measuring benzene in ambient air mentioned in NAAQS i.e A GC – based continuous analyzer and adsorption followed desorption and then analysis done using GC.

### Standard measurement method for benzene in air

There are various methods available for monitoring benzene level in ambient air. These methods involve collecting samples using absorption tubes, canister, passive sampler and subsequently analysing them through gas chromatography with different detector like MS, FID, PID. Additionally, other techniques are employed for sampling and analysis including real-time continuous monitoring systems, portable instruments for direct reading, or other suitable methods depending on the source of pollutants. The selection of monitoring methods for benzene is crucial to ensure accuracy and precision in the results obtained. Each country establishes its own standard limits for exposure and specifies the methods for sampling and analysis. In this section, both national and international standard methods for monitoring of benzene in ambient air has been discussed.

### National air quality measurement standard method

The Bureau of Indian Standards described the Indian standard (Part 11), second revision in 2006, i.e., IS 5182 (Part 11): 2006, which outlines methods for measuring air pollution, specifically Part 11 for Benzene, Toluene, and Xylene (BTX). This standard provides three different methods for sampling and analysing BTX pollutants in ambient air. Method 1 involves active sampling using a portable battery-powered pump with a low-flow controller and activated charcoal tubes or other suitable adsorbents (particle size ranging from 60 to 80 mesh). The sampling flow rate should be maintained at 20-100 (±2 percent) ml/min for ambient air, and carbon disulfide (CS2) is used for desorbing adsorbed pollutants. Analysis is conducted using GC-FID. Method 2 describe the same sampling procedure as Method 1 but employs thermal desorption for desorption, which improves analytical sensitivity. Method 3 utilizes passive diffusive samplers for sampling, with further analysis conducted as described in Method 1 using the GC-FID technique. The detection limit for BTX using GC-FID falls within the sub-ppb range [83].

### International air quality measurement standard method

Benzene has been categorized as carcinogenic to human health by IARC since 1979 [80]. Surprisingly, only 27 % of the total 193 United Nations Member States regulate air quality standards for benzene. A worldwide summary of air quality standards for benzene can be found in the published literature by Sekar et al.

The European standard EN 14662 describes the standard method for sampling procedures and the operating conditions of automated chromatographs used to measure ambient benzene levels. The European Union mandates continuous monitoring of ambient benzene due to its toxic nature. Among all hazardous VOCs, benzene is one of the pollutants regulated in European countries, with an annual limit value of 5 µ g/m³ at 293 K and 101.4 kPa. Over the past decades, many countries have successfully reduced the percentage of benzene in fuel compositions. The standard methods for monitoring benzene levels in the atmosphere were established by Directive 2008/50/EC. In 2005, EN 14662 was published, consisting of five parts (CEN, 2005-1, 2, 3, 4, and 5). All parts of this standard are currently active, but Part 3 of EN 14662 was revised in 2015 [84].

BS EN 14662-1:2005 provides general guidance for the sampling and analysis of ambient benzene. Sampling is conducted using a pump and active carbon as an adsorbent, followed by thermal desorption and analysis via GC [85]. In contrast, Part 2 (BS EN 14662-2:2005) differs in the sampling procedure, where desorption is achieved using a solvent. This method utilizes sample tubes containing approximately 100 mg of activated charcoal, with desorption performed using carbon disulfide and subsequent analysis via gas chromatography [86]. BS EN 14662-3:2015, Part 3 of the European Standard, specifies a semi-continuous measurement method for determining ambient benzene levels through automated sampling and gas chromatograph analysis. This method is applicable to various areas, including rural, urban traffic-oriented, and industrial areas, and can measure up to 50 μg/m³ of benzene in ambient air [84]. Both Parts 4 (BS EN 14662-4:2005) and 5 (BS EN 14662-5:2005) of the European Standard describe methods for measuring benzene concentrations using diffusive samplers for sample collection, but differs by desorption procedures i.e thermal desorption for Part 4 and solvent desorption for Part 5 [87-88].

Occupational Safety and Health Administration Standard 1910.1028, Appendix D, Part No. 1910, outlines sampling and analytical methods for monitoring and measuring benzene. OSHA Laboratory Method 12 provides two analytical approaches for measuring benzene levels in ambient air [89].

1. OSHA Method 12 for Air Samples: This method employs charcoal tubes to adsorb organic vapours present in ambient air using a personal sampling pump, followed by desorption with CS2 and analysis using GC. The detection limit for this method is 0.04 ppm, with a sampling rate ranging from 10 L to 0.2 L/min

2. OSHA Method 12 for Bulk Samples: In this approach, samples are analysed using HPLC (high-performance liquid chromatography). The detection limit for this method is 0.01 % by volume.

The United States Environmental Protection Agency (USEPA) describes two methods, 325A and 325B, for monitoring VOCs from fugitive and area sources. Method 325A explains sampler deployment and VOC sample collection, while Method 325B covers sample preparation and analysis. Both methods are suitable for measuring benzene using GC-FID/MS within a concentration range of approximately 0.5 to 500 µ g/m³ [90-91]. Other US EPA measurement method of VOCs in ambient air given in Table 5.

### Sampling and measurement techniques of benzene

#### Sampling methods

Sampling of benzene in ambient air is very important for assessing air quality and ensuring public health associated due to its exposure in air. There are several methods for sampling of benzene in ambient air, each have their advantages and disadvantages. Here, most commonly sampling divided as active and passive sampling.

Active sampling: A pump is used to sucked a known volume of air through sampling device over a specific period of time. During this process, pollutants present in the air are collected on the sampling medium. The flow rate and sampling time carefully controlled to maintain accuracy. Charcoal sorbent tubes are used most preferably to adsorbed VOCs including benzene. Sorbent tube is analysed by desorption in CS2 or other solvents followed by gas chromatography (https://www.cpcb.nic.in/). In 2023, Poonam Kumari et al find out concentration of BTEX at fuel station [2] and benzene concentration at traffic interjection using active sampling [3]. The health risk associated with VOCs and carbonyl compounds was determined using active sampling in beauty salons [92].

#### Passive sampling

A diffusion sampling tube consists adsorbents are used to reinforce the targeted analytes. Analytes present in the ambient air diffuse through the permeable membrane or are absorbed by the sorbent material within the passive sampler. The rate of diffusion and absorption is determined by the concentration gradient between the air and the sampler, as well as the specific properties of the sampler and the target analytes. After exposure, the sorbent is analyzed using techniques such as thermal desorption coupled with GC-FID or GC-MS. Passive sampling is useful for long-term monitoring and can provide time-weighted average concentration (https://www.cpcb.nic.in/). In last few decades, passive sampler has been mostly used due factor like easy to use, their simplicity and low cost. In 2019 Vallecillos et al. use passive sampler for volatile organic compounds analysis in industrial atmosphere [93]. Recently in 2023, the concentration of pollutants is measured using passive sampling in Brazilian urban areas, the level of benzene exceeded the limit according to the WHO, i.e 1.7 μg m^3^ associated with the probability of leukemia [94].

#### Grab sampling

In grab sampling sample air is taken in a container like polished stainless-steel canisters, bags in a very short periods of time (10-30 seconds). Then analysis of the sample is done using gas chromatography or other suitable techniques. In 2010, Tiwari et al analysed the concentration of VOCs in petrochemical industrial area using active grab sampling [95]. Other details of sampling procedure and with their advantage and disadvantage given in Table 6.

### Measurement techniques

**Figure 5.**
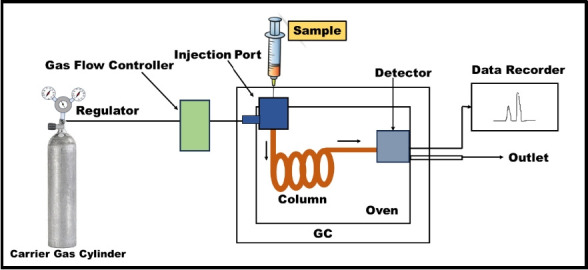
Schematic diagram of Gas chromatography

Various methods are present for determining the concentration of benzene in different environments, including ambient air, workplaces, water, food, fuel stations, and soil. Gas chromatography (GC) is a commonly used technique for separation, employing different detectors like FID, MS, or PID. The limit of detection for GC-FID/PID ranges from low parts per billion (ppb) to parts per trillion (ppt) levels. GC-MS considered more reliable and accurate for separation of similar GC elution characteristics. The limit of detection for benzene in ambient air has been quantified at sub-ppb levels using GC-MS [46]. A schematic diagram of GC is given in Fig. 5.

In the literature, GC-FID is predominantly used. In 2019, Garg et al. utilized activated charcoal tubes to collect BTEX samples in ambient air, subsequently analyzed using GC-FID [78]. Sousa et al studied benzene levels in indoor and outdoor environments using the GC-FID technique, with tandem mass spectrometric detection (MS/MS) used to confirm benzene in ambient air [96]. Bayatian et al reported a new method involving a sampling needle trap device (NTD) and analysis performed using GC-FID, with benzene level measured in the range of 0.15 to 1.2 ppm [97]. Kumari et al reported benzene concentrations at fuel stations and traffic intersections, with sampling conducted using charcoal sorbent tubes and subsequent analysis using GC-FID [2-3]. Tabatabaei et al examined BTEX concentrations emitted by Hookah smoke in indoor air, with sampling carried out using charcoal adsorbent tubes and subsequent analysis using GC-FID [79]. Disadvantages of gas chromatography include longer analysis times and limitations in real-time monitoring or lack of direct quantification of analytes [98].

Proton transfer reaction mass spectrometry is a technique used for real-time monitoring of pollutants in the atmosphere. It is employed for measurement of VOCs in atmosphere using low chemical energy. The separation of volatile organic compounds occurs based on their mass/charge ratio. Literature [99-101] reported this technique for separating VOCs, including benzene in the atmosphere. The advantages of PTR-MS include short time resolution (sub-minutes), suitability for long-term continuous measurement, and a low limit of detection. A disadvantage of this technique is its inability to distinguish between pollutants with the same nominal mass [98].

The BTEX Analyzer is a real-time monitoring instrument for pollutants, capable of analyzing BTEX concentrations simultaneously. Data obtained can be transferred via modem. However, a drawback of this technique is its high cost, as well as the need for carrier gas to operate the instrument. APCI-MS means atmospheric pressure chemical ionization mass spectrometry, offers high ionization efficiency and sensitivity, allowing for direct sampling. However, it is not suggested for measurement of BTEX at low concentrations due to the formation of water clusters and it is very expensive. Many techniques used for measurement of volatile organic compounds including benzene reported by Sylwia et al. [102].

### Level of benzene in ambient air

**Figure 6.**
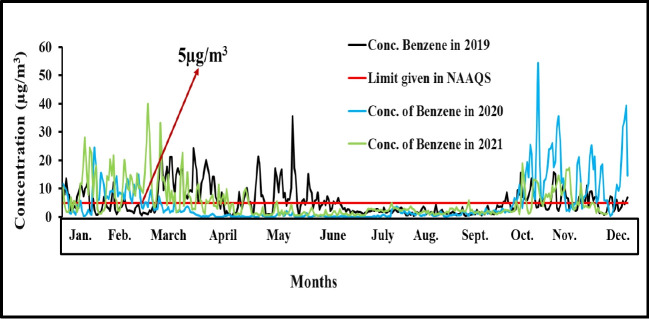
Monthly concentration of benzene from 2019-2021.

Benzene is considered a carcinogenic pollutant according to EPA and WHO guidelines, and it may have numerous adverse health effects on both humans and animals. Due to its detrimental health impacts, continuous monitoring of this pollutant is necessary. Several studies have been conducted in major hotspot areas around the world [2,3,15,21,31,35,47,75, 103-111]. Literature related to observed values of benzene and other VOCs at fuel stations has been published [2, 112-117]. Jafari et al studied atmospheric benzene and its health effects in Tehran megacity from 2010- 2013, with yearly average benzene concentration ranging from 1.84 to 2.57 µ g/m³ [118]. Continuously three-year (24 hrs average) benzene concentration from 2019-2021 was observed through online monitoring station at shadipur New Delhi set by central pollution control board (CPCB). Maximum duration concentration of benzene was exceeding the annual limit given in NAAQS parameter i.e 5 µ g/m³ except lockdown period during corona pandemic shown in Fig 6.

Popitanu et al examined the seasonal impact of BTEX pollutants in Arad City, Romania. BTEX (Benzene, Toluene, Ethylbenzene, Xylene) concentrations were highest in winter and lowest in autumn, with benzene being the most dominant pollutant. The mean value of benzene ranged from 2.47 ± 0.74 µ g/m³ to 18.00 ± 1.32 µ g/m³. The LTCR (Lifetime Cancer Risk) for benzene was higher than the limit set by WHO guidelines during the winter season, reaching 3 × 10⁻⁵, categorized as 'possible cancer risk.' However, during the summer, LTCR for benzene was less than 10⁻⁵, indicating 'probable cancer risk.' The observed level of benzene exceeds the limit i.e 5 µ g/m³ limit set by Directive 2000/69/E.C [103].

Fariba et al studied benzene in a Middle Eastern city and found a concentration of 2.95 µ g/m³, within the range [119]. The mean ILCR for benzene were 6.49 × 10⁻⁷ for infants (0-1 year), 7.21 × 10⁻⁶ for children (2-18 years), and 1.27 × 10⁻⁵ for adults (19-70 years), exceeding the limits set by the US EPA and WHO (1 × 10⁻⁶ - 1 × 10⁻⁵). Various studies on benzene pollutant in ambient air have been conducted in Western urban areas in Europe, North America, and Japan, with concentration ranges provided [120-121].

A study by Garg and Gupta in 2019 investigated benzene concentrations in New Delhi during busy and non-busy hours. The average benzene concentration during busy hours was 12.32 ± 6.69 μg/m³, while during non-busy hours, it was 8.50 ± 5.83 μg/m³ [78]. Srivastava et al conducted a study in Mumbai on VOCs in ambient air, covering various types of areas. The annual mean benzene values with standard deviations were obtained for different locations, including residential areas, commercial areas, industrial areas, traffic intersections, and petrol pumps, ranging from (45.31 ± 18.68) to (539.95 ± 51.20) µ g/m³ [44]. Kashyap et al carried out a study on BTEX concentrations in urban vegetative sites in New Delhi, India, area of his study was SJP (Swarn Jayanti Park), Rohini, and JNU (Jawaharlal Nehru University) and also Yamuna biodiversity park, reporting an average benzene value of 8.98 mg/m³ [107]. Similarly, a study on non-methane hydrocarbons (NMHC) was conducted in India by Kumar et al. (2019) at different locations, with the highest concentration of benzene (67.8 µ g/m³) observed at ITO (Income Tax Office Crossing) [122].

Masih et al studied BTX pollutant concentrations in North India and reported a total BTX concentration of 30.95 µ g/m³ at Gorakhpur in urban and rural areas, with mean benzene values of 12.1 and 7.4 µ g/m³, respectively [123]. Garg et al observed benzene concentrations at six locations in Delhi, with the highest value reaching 58.73 µ g/m³ at Anand Vihar during the winter season. Most locations in Delhi recorded benzene concentrations higher than the 5 µ g/m³ NAAQS standards, highlighting the need for improved air quality measures in urban areas. Concluded that urban atmosphere like in Delhi city, hazardous pollutant like benzene not easily dissipate into ambient air. so, we need to improved our technologies for better air quality like improve fuel quality, traffic management strategies, odd-even policy, growing plants on road sides, planning meeting time to time for pollution management and increase greenery areas in such megacity may result in reduction of such toxic pollutions [111]. In a recent publication, Kumari et al observed benzene concentrations at fuel stations and traffic intersections in Delhi that exceeded the prescribed limits set by NAAQS parameters. The average benzene concentration was found to be (84 ± 40) µ g/m³ at fuel stations, using charcoal sorbent tubes for sampling, with the maximum average value of benzene reaching 33 μg/m³ at traffic intersections [2-3]. Another study in India by Buddhadev Ghosh et al. (2023) studied the spatio-temporal distribution and health risk assessment of BTEX in the urban atmosphere from 2019 to 2022. The average value of benzene was (8.85 ± 4.34) µ g/m³ in Kolkata and (7.25 ± 0.42) µ g/m³ in Howrah, both exceeding the annual limits set by NAAQS for ambient air in India.

Notario et al conducted a study on ambient benzene in metropolitan and industrial areas of Spain from 2014-2017. The yearly average benzene level change from 0.3 μg/m³ to 2.4 μg/m³, with hourly maximum values reaching 112 μg/m³ [124]. Behnami et al investigated the spatiotemporal variation of benzene and other VOCs using charcoal sorbents in Iran, finding a mean benzene value of (29 ± 1.07) μg/m³ [125]. Mostly benzene concentration was found above the yearly average limit i.e 5 μg/m³ in developing countries due to rapidly increase industrialization, urbanization as compare to rural areas. So, we concluded that benzene pollution vary area wise depending on their specific regulatory frameworks, industrial activities, and environmental challenges.

In India, most studies on benzene pollutants have focused on metro cities like Delhi and Kolkata, primarily examining exposure in urban areas. Given the hazardous health and environmental impacts of such pollutants, more research is needed in rural areas, raising awareness among rural communities regarding emissions from sources such as crop burning, wood cooking, furniture, and painting. Monitoring benzene in both rural and urban areas is crucial, especially since a significant portion of the population resides near high-traffic junctions or alongside roads [123]. A literature survey on benzene concentration worldwide given in Table 7.

### Certified reference material for monitoring of benzene in ambient air

A primary reference gas materials or certified reference materials (CRMs) are an important factor to certify the traceability of measurement results. [39-40,130]. The literature [38-39,131-137] reported certified reference material of volatile organic compounds including benzene in different matrix gas. CSIR-NPL national metrology institute (NMI) of India have been developed (2.79 ± 0.015) to (13.64 ± 0.020) μmol/mol benzene in nitrogen calibration gas mixture [38]. Different National Metrology Institute (NMI) of world-wide developed Gravimetric prepared CRM for multicomponent of VOC like BTEX and participate in international inter comparison. Latest last key comparison CCQM-K10.2018 final report was published in 2022. More details about preparation procedure and measurement techniques of reference gas mixture is given in a published review article [138]. Detail description of benzene certified reference material participation by different NMI are given in Table 8 and 9.

### Mitigations strategies

Vehicular emission and fuel vaporization at fuel stations are found to be the main sources based on the results of the emission inventory and reported monitoring data of benzene in ambient air. Priority mitigation strategies should mainly focus on these sources.

• Prefer walking or riding instead of driving nearby workplaces, use public transport instead personal vehicle.

• Promote electric vehicles project and develop smart traffic management controller agencies to reduce jam [139].

• Developing traffic efficient applications for road networks in the smart cities [140].

• Promote public transportation project to encourage residents use buses [141].

• Developing a red alert warning system at monitoring stations whenever high concentration of benzene observed to protect human.

• Choose a fuel-efficient vehicle when replacing new car.

• Proper fuel handling training to be given the employers follow up during loading and unloading the fuel.

• Promote self-service fuel stations.

• Mandate protective gear such as gloves and breathing masks for all employers at fuel station [115].

• Incorporate the features in designing of the fuel stations which will improve the air exchange rate.

Implementation of stringent emission standards for industrial processes and vehicular emissions, urban planning and traffic management, Continuous monitoring and surveillance, Reduction of benzene content in gasoline, green spaces and vegetation, public awareness and control of tobacco uses, Installation of Vapour recovery system at fuel stations, use eco- friendly cleaning products are the key factor to mitigate the benzene emission in air.

Gardening of the toxic filter plants i.e Spider plants, Snake plants, Weeping figs, Peace Lily, Bamboo palms, Boston ferns, Aloe Vera, Dracaenas, English ivy, Janet Craig, Barberton Daisy, Chrysanthemums that absorbed benzene from air.

Continuous monitoring of benzene concentration in ambient air is important for identifying pollution hotspots and assessing the desired output of mitigation measures. Timely accurate measurement results to be provided to policy decision-makers which help them to take prompt appropriate action to enhance the air quality.

## Conclusions

In this article, we have discussed the significance of hazardous pollutant benzene in our environment. There are many complexities and challenges associated with accurate assessment and mitigation of benzene exposure. Attention must be given to this silent killer to ensure the safety of individuals and the environment. The profound impact of benzene on human health, including its association with cancer and other adverse health, reinforces the top priorities of comprehensive mitigation strategies. The concentration of benzene measured at different sites in different countries is found to be higher than the exposure limits in air. Even though standards of benzene are accessible, in most cases they are not capable of safeguarding public health. Metrological traceability plays an important role to ensure the accuracy of the measured data. A very few countries are developing certified reference material of benzene worldwide. In summary, this review article discussed complicated nature of benzene assessment and will contribute to determine the potential risks on populations due to presence of the ambient benzene. The study promotes the monitoring strategies, implementation in air quality monitoring station designed and will help experts in decision making and risk management. The benzene pollution could be controlled by authorities or policy makers (by implementing stringent air quality standard, promoting alternative transportation), researcher (by developing advanced monitoring techniques, case studies to investigate the health effects of benzene exposure on communities), industry stakeholders through partnerships with government agencies, research institutions, and share best practices, data, and resources for addressing benzene pollution effectively. It is essential for researchers, policymakers, and industries to collaborate in a concerted effort to refine measurement methodologies, establish various reference materials, amend exposure limits, and improve mitigation strategies. Only through such collective action we can reduce the risks associated with benzene exposure and protect both individuals and the environment from its harmful effects.

## Figures and Tables

**Table 1. t1-eaht-39-2-e2024012:** Description of benzene

Specification	Nature
Chemical formula	C6H6
Physical form (20°C)	Clear liquid (colourless)
Molecular weight	78.11 g/mol
Nature	Volatile, Colourless, flammable
Vapor pressure (VP)	95.2 mm Hg (25 °C)
Boiling point (BP)	80.1 °C
Melting point (MP)	5.5 °C at 20 °C
Flash Point (0°C)	-11.1 °C
Flammable limits	1.3 - 7.1 %
Conversion factors in gaseous form	1 mg/m^3^ = 0.313 ppm
	1 ppm = 3.19 mg/ m^3^

Source: - Wikipedia

**Table 2.** Brief description of benzene source in ambient air.

no.	Source	Description of sources
1.	Fuel stations	Petrol pumps, gas station, CNG station
2.	Smoke	Cigarette, Biomass burning
3.	Vehicle exhaust	Transport sector, Aeroplane, Road gasoline vehicles
4.	Paints and fragrances	Paint strippers and coating, Aerosol sprays, cleanser and disinfectants, air fresheners,
5.	Office equipment’s	Copier and printer, correction fluids, carbonless paper, permanent markers glues etc
6.	Industries	Chemical industries, crude oil processing, refineries
7.	Natural	Volcanoes and forest fires

**Table 3.** National & International Occupational Exposure limits for Benzene.

Country	TWA 8 h Average (ppm^*^)	STEL 15 Minute average (ppm^*^)
India	0.5	2.5
USA-OSHA	1	5
USA-NIOSH	0.1	1
USA-ACGIH	0.5	2.5
Australia	1	-
France	1	-
Germany		-
Ireland	1	-
Israel	0.5	2.5
Romania	1	-
Republic of Korea	1	5
Sweden	0.5	3
Turkey	1	-
United Kingdom	1	-
Spain	1	-
Denmark	0.5	1
Canada – Ontario	0.5	2.5
Belgium	1	-
European Union	1	-
Netherlands	0.2	-

* Conversion factors: Benzene: 1 ppm = 3.19 mg/m^3^ [49].

**Table 4.** Ambient air quality standard limit of benzene country-wise.

Exposure Period	Value (μg/m^3^)	Countries
Annual	5	India, Lebanon, Russia, South Korean, New Zealand, North America Botswana, Albania
Annual	3	Iraq, Japan
Annual	3.25	Scotland, Northern Ireland
Annual	20	Syria
Annual	10	Vietnam, Morocco, South Africa, Belarus
Annual	2	France
Annual	2-3.5	Malta and Sweden
Annual	1.3	Israel
24 h	100	Russia
20 min	300	Russia
1 h	22	Vietnam
24 h	3.9	Israel
8 h	5	Albania
24 h	40	Belarus
24 h	1000	N. America Cuba

Source: [80].

**Table 5.** USEPA method for measurement of VOCs including benzene in ambient air.

Method	Compound	Sampling and Analysis	Detection Limit
TO -1	VOCs (80 °C to 200 °C)	TENAX-GC Adsorption and GC/MS or GC/FID analysis	0.01-10 ppbv
TO - 14 A	VOCs Non polar	Canister and GC/FID/ECD or GC/MS detection	0.2-25 ppbv
TO - 15	VOCs	Canister and GC/MS analysis	0.2-25 ppbv
TO - 16	VOCs	FTIR open path spectroscopy	25-500 ppbv
TO - 17	VOCs	Multi-bed adsorbent tube followed by GC/MS, FID	0.2-25 ppbv

**Table 6.** Sampling medium and method of benzene in ambient air

Sampling medium	Method	Advantage	Disadvantage
SUMMA Canister	Air is sucked into a SUMMA canister, which contains a vacuum. The benzene present in the ambient air goes into canister.	Canisters can capture a wide range of VOCs, including benzene, and provide accurate results	Canisters are relatively expensive and require careful handling to prevent contamination.
Sorbent tubes	Air is sucked through a sorbent tube filled with an adsorbent material (such as activated charcoal) that captures benzene and other volatile organic compounds (VOCs)	Cost-effective, easy to use, and suitable for personal or area sampling.	Sampling flow rate, time and temperature can affect the accuracy of results obtained.
Tedlar bags	Tedlar bags are designed to collect gas/air samples. It fitted with two components i.e Poly Vinyl Fluoride film and Teflon/PTFE valve fitting.	Portable for on-site use, easy to handle.	Out-gassing of bag material, incorrect analytical results, inconsistency in blank levels.
Passive Diffusion Samplers	A diffusion tube containing an adsorbent material. Pollutants diffuses through a membrane and gets adsorbed onto the material	Low-cost, and suitable for long-term sampling. Obtained time-weighted average concentrations of pollutants.	Less accurate than active sampling methods. Results might be affected by weather conditions such rain, wind speed etc.
Real-time monitoring instruments	Online monitoring analyser attached with photoionization detectors (PID) or flame ionization detectors (FID) can provide real-time measurements of benzene concentrations in ambient air.	Instantaneous results, suitable for on-site monitoring and industrial applications.	Large size instruments, heavy weight and extraordinary expensiveness. Also, calibration and maintenance are crucial for accuracy.

**Table 7.** Concentration of benzene worldwide

City	Benzene (μg/m^3^)	Study area	Sampling method/ Measurement Technique	Reference
Delhi (India)	2 - 43	Shadipur, Traffic junction	Coconut charcoal sorbent tubes (SKC, Anasorb CSC, sorbent; extraction with acetone/ GC-FID; Agilent 6890N	[3]
Tehran (Iran)	5200	Fuel station	Personal samplings pump using activated charcoal tube, GC-FID	[114]
Delhi (India)	142.2	IIT Delhi & UP Boarder, Traffic junction	ORBO™-32 Charcoal tube/GC-FID; Nucon 5700 gas chromatography	[126]
Ardabil (Iran)	1690	Fuel station	SKC personal sampling pumps, Coconut charcoal sorbent tubes, GC-FID	[113]
Delhi (India)	1.98 -10.26	Shahdara, Traffic-Congested Area	Charcoal tubes (ORBOTM32)/GC-FID	[111]
USA	2900	Fuel station	Passive monitor, Tenax adsorbent, GC/FID	[127]
Leon, Guanajuato (Mexico)	1.96	Traffic, Industrial Urban area	Active sampling using Charcoal sorbent tubes, 1.5 h, 200 mL min−1, CS2 solvent for desorption/ GC-FID	[106]
Bangkok (Thailand)	590 ± 107	Fuel station	Charcoal glass tube, personal air pump, GC-FID	[128]
Kolkata (India)	24.97 - 79.18	Traffic, Industrial Urban area	Active sampling using Charcoal sorbent tubes, 100 mL min−1, 6 h, CS2 desorption solvent/ GC-FID	[129]
Uttar Pradesh (India)	17 - 29	Fuel station	Real-time measurements using PID detector	[4]
Iran	2 - 108	Industrial and local road traffic area	Active sampling using activated carbon ,2 h, SKC Inc., England, model 222-3	[105]
M.P (India)	2 - 12	Fuel station	Benzene sampler, GC-FID	[112]
Arad City (Romania)	18.00 ± 1.32	High-density traffic area	Stainless steel tubes, SKC 1003, GC-MS attached with a thermal desorption system.	[103]
Delhi (India)	6406	Fuel station	Sampling pump using Charcoal tube, GC-FID	[115]
Delhi (India)	48 - 110	Traffic area, industrial area	Diffusive sampling using coconut shell charcoal, Thomas Baker Pvt. Ltd. for one week. CS2 desorption/GC-FID (PerkinElmer Auto-XL)	[37]
Delhi (India)	112	Fuel station	Sampling pump using charcoal/Tenax sorbent tubes, GC-FID	[2]
Tehran	1.84 - 2.57	Air quality monitoring stations	Gas chromatograph (GC) VOC analyzer	[118]
Shiraz (Iran)	2.95	Population density traffic area	Passive samplers (Radiello RAD 130), GC-MS	[119]

**Table 8.** Details of certified reference material of benzene developed worldwide.

NMI	Country	Techniques	Gravimetric amount-of substance fraction (nmol/mol)	Standard uncertainty (k=1) (nmol/mol)
KRISS	Korea	GC-FID	5.03	0.098
LNE	France	GC-FID	5.09	0.050
NIST	USA	GC/FID/preconcentration	5.47	0.042
NIMSA	South Africa	Cryogenic pre-concentration coupled with GC	5.02	0.063
NPL	United Kingdom	GC-FID	5.00	0.050
VNIIM	Russia	Chromato-mass-spectrometer	4.97	0.055
VSL	Netherlands	GC/FID	5.00	0.050
METAS	Switzerland	ATD-GC-FID	6.21	0.360
UBA	Germany	GC Clarus 680 GL and Turbomatrix 300 a	5.09	0.020
CHMI	Czech Republic	GC/ FID Preconcetration	5.15	0.157

Source: [133]

**Table 9.** Details of component transfer method and column used for analysis.

NMI	Component transfer method	Column	Cylinder Pressure
KRISS	Binary-micro -syringes	-	9.3 Mpa
LNE	Binary-syringes	CP-xylene capillary column	125 bars
NIST	Binary-capillary tubes	-	1500 psi
NIMSA	Syringe injection	60 m × 0.32 mm × 0.5 mm AT_WAX (heliflex)	132 bar.
NPL	Binary 3-way transfer vessels	60 m ×0.32 um, df = 1 um RTX-Wax (Restek corporation).	100 bar
VNIIM	-	Restek Stabilwax (30m × 0.25mmID × 0.25µm)	10.5 MPa
VSL	Liquid mixture; transfer line	CPWAX 52CB (60m× 0.32mm×0.25µm).	125 bar
METAS	Permeation and dynamic dilution using adsorption tube	Stabilwax, Restek	90 bars
UBA	Capillary tubes	-	-
CHMI	-	DB-1 (60m × 320µm × 1 µm)	11.0-10.4 MPa
		HP-Plot Al2O3S (50m × 320µm × 8 µm)	

Source: [133]
